# *Pseudomonas aeruginosa* Rhamnolipids Produced by Andiroba (*Carapa guianensis* Aubl.) (Sapindales: Meliaceae) Biomass Waste from Amazon: A Potential Weapon Against *Aedes aegypti* L. (Diptera: Culicidae)

**DOI:** 10.3390/molecules30030618

**Published:** 2025-01-31

**Authors:** Giulian César da Silva Sá, Pedro Vitor Vale Bezerra, Evelly Oliveira Ramos, Alexandre Orsato, Karoline Leite, Alan Moura Feio, Lucas Mariano Siqueira Pimentel, Joane de Almeida Alves, Glenda Soares Gomes, Pamela Dias Rodrigues, Cristina M. Quintella, Sinara Pereira Fragoso, Emilly Cruz da Silva, Adriana Ferreira Uchôa, Sidnei Cerqueira dos Santos

**Affiliations:** 1 Laboratório de Bioensaios e Bioprocessos, Instituto de Estudos em Biológicas e Saúde, Universidade Federal do Sul e Sudeste do Pará (Unifesspa), Marabá 68500-000, PA, Brazil; evelly.ramos@unifesspa.edu.br (E.O.R.); alan.moura@unifesspa.edu.br (A.M.F.); lucas.pimentel@unifesspa.edu.br (L.M.S.P.); joaneaa@unifesspa.edu.br (J.d.A.A.); glenda.soares@unifesspa.edu.br (G.S.G.); 2 Laboratório de Proteomas, Instituto de Medicina Tropical do Rio Grande do Norte, Universidade Federal do Rio Grande do Norte (UFRN), Natal 59078-970, RN, Brazil; pedro.vale.080@ufrn.edu.br (P.V.V.B.); adriana.uchoa@ufrn.br (A.F.U.); 3 Laboratório de Síntese de Moléculas Medicinais, Departamento de Química, Universidade Estadual de Londrina (UEL), Londrina 86057-970, PR, Brazil; orsato@uel.br (A.O.); karoline.leite@uel.br (K.L.); 4 Laboratório de Cinética e Dinâmica Molecular, Departamento de Química Inorgânica e Geral, Universidade Federal da Bahia (UFBA), Salvador 40170-115, BA, Brazil; pamelarodrigues.ufba@gmail.com (P.D.R.); cris5000tina@gmail.com (C.M.Q.); 5 Laboratório de Tecnologia de Alimentos, Universidade Federal da Paraíba (UFPB), Centro de Tecnologia, João Pessoa 58051-900, PB, Brazil; sinarafragoso@hotmail.com; 6 Laboratório de Bioensaios e Bioprocessos, Instituto de Estudos em Saúde e Biológicas, Universidade Federal do Sul e Sudeste do Pará (Unifesspa), Marabá 68500-000, PA, Brazil; emillycruzds@gmail.com

**Keywords:** biodiversity, biosurfactant, microbial, mosquito control

## Abstract

Rhamnolipids, biosurfactants synthesized from natural resources, demonstrate significant applications, including notable insecticidal efficacy against *Aedes aegypti* L., the primary vector for numerous arboviruses. The global spread of *A. aegypti* poses substantial public health challenges, requiring innovative and sustainable control strategies. This research investigates the use of andiroba (*Carapa guianensis* Aubl.) biomass waste as a substrate for synthesizing a rhamnolipid biosurfactant (BSAW) produced by *Pseudomonas aeruginosa* and evaluates its insecticidal activity against *A. aegypti*. The findings indicate a biosurfactant yield of 4.42 mg mL^−1^, alongside an emulsification index approaching 60%. BSAW successfully reduced both surface and interfacial tensions to below 30 mN/m and 4 mN/m, respectively. Characterization revealed that BSAW is a di-rhamnolipid, consisting of two rhamnose units covalently linked to a saturated C_10_ fatty acid chain. At a concentration of 1.0 mg mL^−1^, BSAW exhibited notable larvicidal activity, leading to structural impairments and cellular dysfunctions in *A. aegypti* larvae while also disrupting their associated bacterial microbiota. Moreover, BSAW effectively deterred oviposition in adult mosquitoes. These findings underscore BSAW’s potential to compromise various developmental stages of *A. aegypti*, supporting integrated arbovirus management approaches. Furthermore, this research emphasizes the feasibility of utilizing agro-industrial waste as substrates for microbial rhamnolipid production.

## 1. Introduction

Natural products are essential to numerous biological processes, offering significant benefits across various sectors, including medicine and agriculture. These bioactive compounds, derived from living organisms, have been instrumental in developing novel pharmaceuticals, advancing in sustainable agricultural practices, and driving progress in biotechnology [1]. The synthesis of natural products provides a benchmark for assessing innovative methodologies and understanding the functional applicability of bioactive compounds [2].

Recent investigations by Siqueira et al. [3] and Silva et al. [4] have focused on the biosynthesis of rhamnolipid biosurfactants, which exhibit notable insecticidal properties. Rhamnolipids, a subclass of biosurfactants, are biocompatible amphiphilic molecules that effectively reduce surface and interfacial tensions among immiscible substances [5]. These bioactive compounds are predominantly produced by *Pseudomonas aeruginosa*, a versatile Gram-negative bacterium capable of utilizing alternative substrates for growth [6].

Exploring alternative substrates for rhamnolipid production offers a promising approach to enhance the sustainability of biosurfactant production. The use of andiroba (*Carapa guianensis* Aubl.) waste has emerged as a viable strategy for this purpose. Andiroba, a term derived from Tupi-Guarani, refers to a tree belonging to the Meliaceae family and is abundant in the Amazon region [7]. The use of andiroba waste as a substrate for producing insecticidal rhamnolipids promotes sustainability in biosurfactant production while addressing the significant waste management challenges of the andiroba processing industry. In Belém, the capital of Pará state in Brazil, approximately 200 tons of andiroba waste are produced annually, highlighting the significant potential for valorizing these byproducts [8,9]. Several initiatives have demonstrated the successful repurposing of andiroba waste, particularly by traditional Amazonian communities producing therapeutic soaps and insecticidal candles [10].

The increasing prevalence of arbovirus epidemics, including dengue, Zika, and chikungunya, presents growing public health challenges, especially in tropical regions like Brazil [11]. Arbovirus outbreaks have also been documented in temperate regions, such as the West Nile virus epidemic in the United States in 1999 and the chikungunya outbreak in Italy in 2007, which subsequently spread to Greece and Romania in 2010. During this period, local dengue transmission was also documented in France and Croatia [12].

The *Aedes aegypti* L. mosquito, the primary vector for these diseases, demonstrates remarkable adaptability to urban environments [13]. Current control strategies for *A. aegypti* populations primarily rely on chemical insecticides. However, this approach has resulted in the emergence of resistant strains, diminishing control efficacy and posing risks to non-target organisms, including humans [12,14,15]. This scenario underscores the urgent need for innovative and safer vector control strategies.

Research on rhamnolipids, particularly concerning their insecticidal properties, offers innovative solutions to challenges posed by arbovirus outbreaks. The use of residual biomass of andiroba contributes to waste reduction while providing a sustainable approach to synthesizing bioactive compounds. Investigating the effects of rhamnolipids on different developmental stages of *A. aegypti* can enhance our understanding of their potential. This knowledge may support the development of novel vector control strategies.

The production of rhamnolipids from andiroba waste represents a critical intersection of biotechnology, waste management, and public health. This study examines andiroba biomass waste as a substrate for rhamnolipid biosynthesis by *P. aeruginosa* and evaluates its insecticidal activity against *A. aegypti*.

## 2. Results

### 2.1. BSAW Production

The biosurfactant, referred to as BSAW (biosurfactant from andiroba wastes), was successfully produced by *Pseudomonas aeruginosa* BM02, utilizing andiroba biomass waste as the sole nutrient source. With an extraction yield of 4.42 mg mL^−1^, BSAW exhibited notable physicochemical properties. As shown in Table 1, BSAW displayed an emulsifying activity of up to 60% and a remarkable reduction in surface and interfacial tensions to below 30 mN/m and 4 mN/m, respectively.

A comprehensive investigation of the proximate composition of andiroba biomass waste revealed a complex matrix with high lipid content and substantial carbohydrate fractions, as shown in Table 2. These findings underscore the potential of andiroba biomass waste as a substrate for producing value-added biomolecules, such as rhamnolipids.

### 2.2. Structural Characterization of BSAW

**Figure 1:**
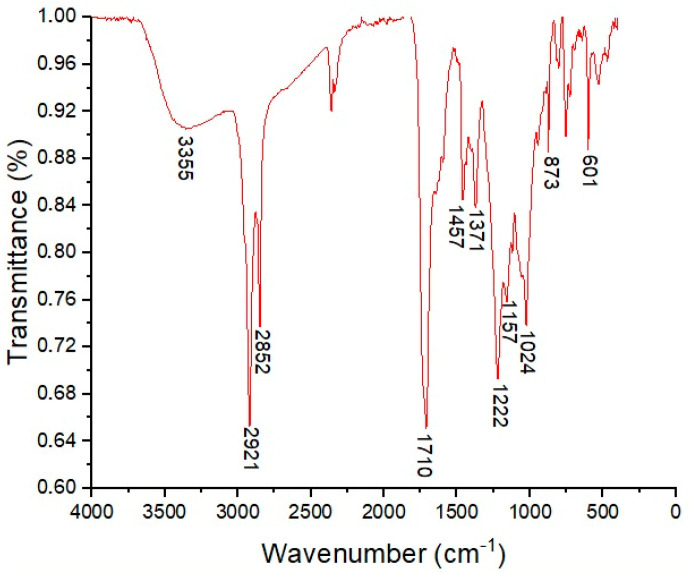
Infrared absorption spectrum (FT-IR; 4000–400 cm^−1^) of biosurfactant (BSAW) produced by *Pseudomonas aeruginosa* BM02.

Fourier-Transform Infrared Spectroscopy (FT-IR) was used to elucidate the structural features of BSAW. The key absorption bands at 3355, 2921, 2852, 1710, and 1024 cm^−1^ (Figure 1) indicate that BSAW has a chemical structure characteristic of rhamnolipids. The broad band at 3355 cm^−1^ corresponds to O-H stretching vibrations from the rhamnose hydroxyl groups. The signals at 2921 and 2852 cm^−1^ correspond to aliphatic C-H bond stretching vibrations, while the peak near 1710 cm^−1^ peak is attributed to C=O bond stretching vibrations in ester functionalities and possibly carboxylic groups of fatty acids. The absorption bands covering 1300 to 1000 cm^−1^ suggest C-O bond stretching in the hydroxyl or ester groups. Furthermore, the peak at 1707 cm^−1^ may represent the C=O stretching of carboxylic groups in fatty acids. The pronounced band at 1024 cm^−1^ correlates with vibrations of the C-O-C groups typical of cyclic carbohydrate structures, confirming the presence of rhamnose units.

Subsequent analyses using Electrospray Ionization Mass Spectrometry (ESI-MS) were performed to examine the lipid side chains and structural homologs of BSAW (Figure S1). The prominent ion at 479 *m/z* indicates that BSAW is a di-rhamnolipid (two rhamnose units) linked to a C_10_ fatty acid chain, with minor proportions of other rhamnolipid variants also likely present.

### 2.3. Larvicidal Effects of BSAW

BSAW’s bioactivity against *A. aegypti* larvae was assessed, revealing its effectiveness as a larvicidal agent, with mortality rates dependent on the exposure duration and the rhamnolipid concentration. The lethal concentration (LC_50_) was determined as 1.0 mg mL^−1^ at 48 h post-exposure (Table 3).

**Figure 2:**
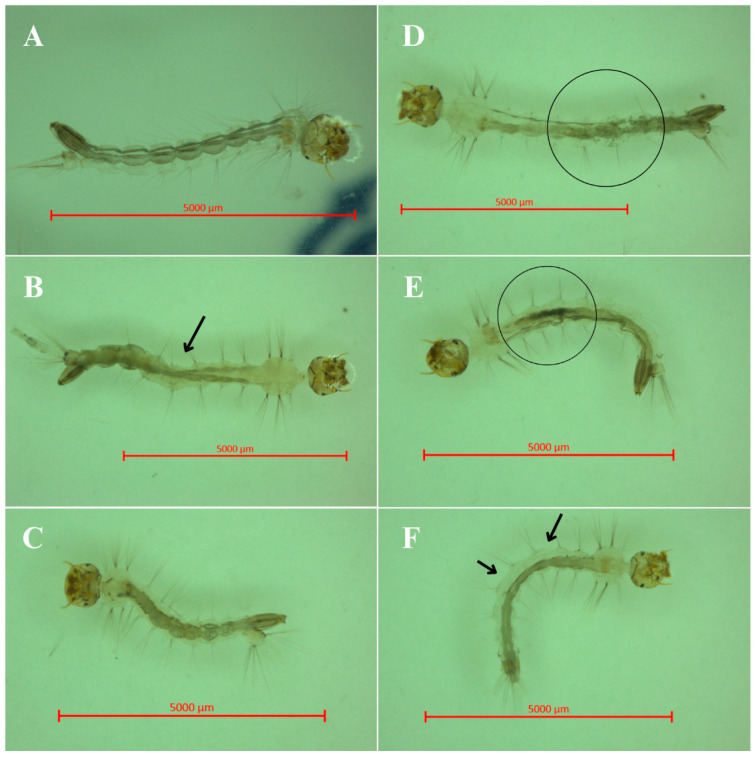
Morphological alterations in BSAW (biosurfactant from andiroba wastes)-treated larvae. (**A**) Control larva; (**B**–**F**) BSAW-treated larvae; (**B**) Exoskeleton deformation; (**C**) Larval shortening; (**D**) Intestinal disintegration; (**E**) Melanization; (**F**) Peritrophic membrane detachment. Circles and arrows highlight observed changes.

The observed larval mortality is likely due to physiological impairments, as indicated by the morphological alterations in the BSAW-treated larvae, as shown in Figure 2. Notable exoskeletal deformities and structural alterations, including alterations to the respiratory siphon and peritrophic membrane, were observed. These deformities likely compromise respiration and nutrient absorption, ultimately contributing to larval mortality. Other notable alterations included melanization within the mid-intestine, disintegration at the terminal ends, and granule formation near the respiratory siphon. Larval length measurements revealed a significant reduction in treated specimens, with an average length of 3.6 mm compared to 4.8 mm for the control larvae, emphasizing BSAW’s impact on larval growth and development.

### 2.4. Impacts of BSAW on the Gut Microbiota Homeostasis

**Figure 3:**
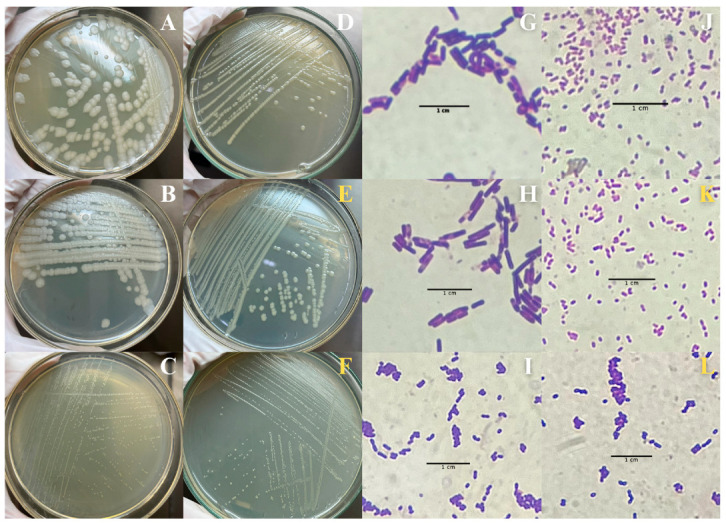
Bacterial composition within the intestinal homogenate of *Aedes aegypti* larvae. (**A**–**D**,**G**–**J**): bacterial composition within the intestinal homogenate of control larvae, in white tones; (**E**,**F**,**K**,**L**): BSAW (biosurfactant from andiroba wastes)-resistant bacterial, in yellow tones.

In addition to the morphological alterations, the effects of BSAW on the intestinal microbiota of the *A. aegypti* larvae were assessed at a concentration corresponding to the determined LC_50_ (1.0 mg mL^−1^). Initial characterizations of the bacterial composition within the intestinal homogenate of the control larvae revealed the presence of three Gram-positive (Figure 3A–C,G–I) and one Gram-negative (Figure 3D,J) bacterial colonies. These colonies exhibited distinct morphological characteristics, with none surpassing 3 mm in diameter. The disruption of these microbial communities in the BSAW-treated larvae adversely affects larval health and survival; two colonies exhibited susceptibility to the rhamnolipids and were absent in subsequent characterization assays. Conversely, two BSAW-resistant bacterial colonies (Figure 3E,F,K,L) showed similarities to previously identified colonies (Figure 3C,D,I,J), indicating potential shared identities. Comprehensive details regarding the characterization of these colonies are provided in the Supplementary Materials (see Table S1).

### 2.5. Effects of BSAW on Oviposition Behavior

In addition to its larvicidal effects, BSAW (1.0 mg mL^−1^) significantly impacted the oviposition behavior of adult *A. aegypti*. During the experimental period, 665 eggs were recorded, with 41 eggs located in the BSAW-containing traps and 624 eggs in the control traps. The oviposition deterrent index (ODI), approximately −80%, supports BSAW’s effectiveness as an oviposition inhibitor for female adults (Table 4). This significant deterrent effect suggests that BSAW could play a key role in integrated insect management strategies aimed at reducing mosquito populations and controlling vector-borne diseases.

## 3. Discussion

The nutritional composition of the andiroba biomass plays a key role in the production, yield, and physicochemical properties of BSAW. This finding supports the previous studies, particularly Zuo et al. [16], highlighting the importance of substrate nutrients in the metabolism of biosurfactant-producing bacteria. The BSAW extraction yield of 4.42 mg mL^−1^ significantly exceeds the previous yield of 2.28 mg mL^−1^, achieved with glycerol as a substrate using the same bacterial strain, while maintaining comparable emulsifying and surface-active properties [6]. While modestly lower than the 5.5 mg mL^−1^ reported by Dobler et al. [17] for glycerol-based production, this yield is still significant. Although glycerol is a widely used, cost-effective substrate, its byproducts can be toxic to cells, disrupting nutrient flow and compromising enzymatic stability [18].

The high lipid concentration in the andiroba biomass is a key factor in BSAW production. Noordman and Janssen [19] proposed that *P. aeruginosa* uses an energy-dependent mechanism to uptake the hydrophobic compounds required for biosurfactant production. Furthermore, nutrient availability directly influences gene regulation related to the metabolic activities of *P. aeruginosa* [20]. A detailed examination of the nutritional components of andiroba biomass waste, especially its lipid content, could offer valuable insights for optimizing biomass use and improving BSAW production and physicochemical properties.

The structural characterization of BSAW suggests it is composed of di-rhamnolipid, with two rhamnose units linked to a C_10_ fatty acid chain, consistent with the previous findings [6,21]. Rhamnolipids consist of a hydrophilic domain of one or two L-rhamnoses linked to a hydrophobic counterpart, typically comprising one or two hydroxy fatty acids [22]. The rhamnolipids in BSAW closely resemble those produced by *P. aeruginosa*, with hydrophobic fractions varying in chain length from 8 to 16 carbon atoms [23].

Rhamnolipids are known for their ecological sustainability, low toxicity, high biocompatibility, and broad potential applications [23]. This study highlights the insecticidal efficacy of BSAW against the *A. aegypti* larvae, supporting findings by Kim et al. [24] on the insecticidal effects of rhamnolipids produced by the *Pseudomonas* species against peach aphids (*Myzus persicae* S.). The observed mortality is attributed to the predominant component, di-rhamnolipid. Furthermore, Kamal et al. [25] documented similar dose-dependent insecticidal activity of *Pseudomonas* di-rhamnolipids against cotton caterpillars (*Spodoptera litura* F.) and cereal weevils (*Rhyzopertha dominica* F.). Collectively, these findings support the idea that rhamnolipids, including BSAW, can negatively impact insect development.

Tracheal respiration in *A. aegypti* larvae is facilitated by a respiratory siphon at their posterior end, which plays a crucial role in nutrient filtration and sustenance [26]. The significant morphological damage observed in the BSAW-treated larvae suggests that the rhamnolipids may contribute to the disintegration of terminal structures and granule formation near to the siphon. Silva et al. [4] suggested that damage to the structural integrity and proper positioning of the siphon could explain the mortality in the BSAW-treated larvae, as this structure is crucial for respiratory efficiency.

Furthermore, the foundational principles established by Kim et al. [24], Kamal et al. [25], and Silva et al. [4] help to explain the additional morphological alterations observed in this study. Exposure to BSAW caused structural damage to the larvae’s exoskeletons, which may hinder mobility and increase their vulnerability to predation and human interventions. The significant reduction in larval length points to physiological stress, likely resulting from impaired feeding and growth processes [27].

Signs of melanization in the mid-intestines, coupled with the detachment of the peritrophic membrane and terminal ends, suggest a severe disruption of digestive processes, leading to nutrient malabsorption [28]. Furthermore, the exposure to BSAW rhamnolipids seems to compromise vital cuticular waxes and intersegmental membranes, potentially leading to dehydration, separation of cellular components, and eventual larval mortality. Additionally, Al-Tahhan et al. [29] proposed that rhamnolipids may influence gene regulation related to cell membrane constituents, promoting their adsorption into mosquito cell membranes. This adsorption process may lead to the formation of transmembrane pores, causing the leakage of cellular contents.

Beyond the observable morphological changes, BSAW also significantly impacts the intestinal microbiota of the *A. aegypti* larvae. The microbiota of *A. aegypti* consists of both resident and transient microorganisms, with diversity and composition hifting throughout the mosquito’s life cycle, influenced by dietary sources [30]. Our examination of the intestinal bacterial composition in the *A. aegypti* larvae reveals that the insecticidal effects of BSAW extend to the microbial community. This finding supports the previous research showing that alterations in the intestinal microbiota can negatively affect the survival and fitness of the *Aedes* species [31]. The observed differences in community structure, alongside the morphological alterations in the BSAW-treated larvae, highlight the influence of rhamnolipids on larval nutrition and microbiota, consistent with the prior studies [32].

A proposed mode of action for the larvicidal effects of BSAW suggests that, upon ingestion by the *A. aegypti* larvae, the interactions between the rhamnolipids and the intestinal barriers lead to structural and functional impairments. These interactions likely cause significant morphological, physiological, and microbiological disruptions. Impairment of the peritrophic membrane, combined with alterations in microbial composition, can severely affect the nutritional status of larvae, contributing to increased mortality rates. Further investigation into the adsorption mechanisms of BSAW rhamnolipids on mosquito biological membranes could enhance our understanding of their larvicidal effects, offering valuable insights for targeted interventions.

These findings also underscore the profound implications of disruptions in the larval microbial composition for adult mosquitoes. Both larvae and adult mosquitoes harbor distinct bacterial communities in their digestive tracts. The transstadial transmission of microbiota from larvae to adults may influence susceptibility to arboviruses, impacting mosquito fecundity and longevity [33]. Although the effects of BSAW on adult mosquito microbiota were not directly investigated in this study, the potential of BSAW rhamnolipids as effective oviposition deterrents for *A. aegypti* females is noteworthy. Prior research [4] suggested that the unique odor of rhamnolipids is detected by adult *A. aegypti*, offering a plausible explanation for the observed deterrent effects.

The promising results observed in laboratory conditions pave the way for testing BSAW in field settings. Strategically applying BSAW in environments favorable for *A. aegypti* oviposition could offer an innovative method for controlling mosquito populations. The presence of rhamnolipids may alter the chemical perception of adult females, inhibiting their egg-laying behavior. Even if females were to oviposit in BSAW-treated environments, the larvicidal properties of rhamnolipids could effectively suppress the development of immature insects. This dual action of BSAW highlights its considerable potential for the sustainable management of *A. aegypti* populations.

However, implementing BSAW in natural ecosystems requires careful consideration of the potential environmental impacts associated with rhamnolipid use. Despite its sustainable and biodegradable characteristics, it remains essential to understand BSAW’s effects on non-target organisms and overall ecosystem dynamics.

## 4. Materials and Methods

### 4.1. Ethics Approval

No unexpected or unusually high safety hazards were encountered that would necessitate approval from an ethics committee.

### 4.2. Biological Materials

The rhamnolipid-producing bacterium, identified as *Pseudomonas aeruginosa* BM02 (GenBank OP410927.1) by Santos et al. [6], was isolated from superficial soil within a mining area in Pará, Brazil. This strain is duly registered with the Brazilian System for Management of Genetic Heritage and Associated Traditional Knowledge (SisGen) under registration number A4DA401. The biomass waste derived from andiroba (*Carapa guianensis*) seed oil extraction was provided in its raw, unrefined state by the Praialta and Piranheira Agro-Extractive Settlement Project, located in Nova Ipixuna, Pará, Brazil (SisGen registration number A27D6F4).

The *Aedes aegypti* mosquitoes were sourced from a heterogeneous colony maintained at the Laboratory of Entomology at the Universidade Federal do Rio Grande do Norte (UFRN) in Natal, Rio Grande do Norte, Brazil. Adult mosquitoes were collected from various municipalities throughout the state of Rio Grande do Norte and maintained in breeding cages at a constant temperature of 28 °C with a natural photoperiod. The mosquitoes were fed a 10% sugary solution, and female mosquitoes were provided a blood meal from a hamster (*Mesocricetus auratus*) every 48 h. After receiving a blood meal, the females were transferred to separate cages for oviposition, allowing for the subsequent collection of individuals for bioassays and colony maintenance under controlled laboratory conditions.

### 4.3. Proximate Characterization of Andiroba Biomass Waste

The proximate analysis of andiroba biomass waste was conducted in triplicate (technical replicates) following the methodologies established by the Association of Official Analytical Chemists (AOAC). Moisture and ash contents were quantified according to AOAC methods 950.46 [34] and 923.03 [35], respectively. The crude protein content was determined using the Kjeldahl method [36], incorporating a conversion factor of 5.75. Total lipid content was assessed using the method outlined by Bligh and Dyer [37], while the total carbohydrate content was calculated using the difference from the overall nutritional composition [38].

### 4.4. BSAW Obtaining

Biosurfactant production was carried out through liquid fermentation in Erlenmeyer flasks containing a vegetable saline medium (VSM), composed of K_2_HPO_4_, KH_2_PO_4_, (NH_4_)_2_SO_4_, and MgSO_4_·7H_2_O. The medium was adjusted to a pH of 7.0 and enriched with andiroba biomass waste at the optimal concentrations for cultivation. This formulation is protected under industrial confidentiality, with an Invention Patent (BR1020230146740) filed with the Brazilian Institute of Industrial Property.

A bacterial inoculum of 5% (with an optical density at 600 nm ranging from 0.6 to 0.8, measured using a Bel V-M5 Visible Spectrophotometer, Biovera, Wildberg, Germany), previously cultured in Luria Bertani broth (Kasvi), was transferred to the VSM-containing flasks. The flasks were incubated in an orbital shaker (SL-22, Solab, Piracicaba, SP, Brazil) at 180 rpm and 30 °C for 9 days. After fermentation, the culture was centrifuged (SL-700, Solab, Aberdeen, UK; 4500 rpm, 25 °C for 15 min) to yield a cell-free broth, which was then acidified to pH 2.0. A second centrifugation step (4000 rpm, 4 °C for 10 min) was performed, and the rhamnolipid biosurfactant was extracted using a chloroform–methanol solution (3:1, *v*/*v*). The extracted biosurfactant was designated as BSAW, which stands for “biosurfactant from andiroba wastes”. BSAW was concentrated using a rotary evaporator (LGI-52CS-1, Scientific, São Paulo, SP, Brazil) and dried in a circulated air oven until a constant mass was achieved [39].

### 4.5. BSAW Characterization

The characterization of BSAW involved a series of analytical assessments to elucidate its functional properties. The emulsifying activity was measured using the emulsification index, as described by Santos et al. [6], where an emulsification index value of ≥50% indicates active emulsifying properties. Surface and interfacial tension measurements were conducted using a tensiometer (Oca15 plus, Data Physics, Filderstadt, Germany) at 25 °C, employing the hanging drop method, which was integrated with an automatic video imaging system and the Oca 10/Oca 20 software [40].

Structural characterization of BSAW was performed using Fourier-Transform Infrared Spectroscopy (FT-IR) with a Vertex 70 model (Bruker, Billerica, MA, USA), equipped with a Platinum ATR reflectance accessory. Spectra were recorded in the range of 4000 to 400 cm^−1^ and analyzed using OriginPro 8.0 software. Electrospray Ionization Mass Spectrometry (ESI-MS) was conducted using an ultra-high-resolution QTOF mass spectrometer (COMPACT model, Bruker Daltonics, Billerica, MA, USA), with direct injection techniques. Mass spectra were acquired in negative ion mode, covering a mass range from 0 to 1000 *m/z*. Analytical parameters included a capillary voltage of 3.8 kV, nitrogen as the gaseous phase with a flow of 4.0 L min^−1^, a gas temperature of 200 °C, and an ion energy of 5.0 eV.

### 4.6. Insecticide Activity

To assess the insecticidal properties of BSAW, a series of bioassays were performed in triplicates, with three biological replicates for each assay.

#### 4.6.1. Larvicidal Trials

Larvicidal assays followed the guidelines established by the World Health Organization for natural insecticides [41]. A total of 20 fourth-instar larvae (L4) of *A. aegypti* were randomly placed in glass beakers containing 20 mL of BSAW, with concentrations ranging from 1.0 to 0.01 mg mL^−1^, alongside dechlorinated water as a control. Larval survival was monitored at 24 and 48 h of incubation; larvae that exhibited no response to mechanical stimuli were classified as deceased. Morphological alterations in the larvae were documented using photographs captured with a Zeiss (Oberkochen, Germany) Stemi 508 stereo microscope. The lethal concentration (LC) was calculated and expressed in mg mL^−1^ based on the dry weight of BSAW, using Probit analysis for dose–mortality determination, with the results presented alongside a 95% confidence interval.

#### 4.6.2. Oviposition Deterrence Trials

To evaluate the effects of BSAW on oviposition behavior, five gravid female *A. aegypti* were introduced into experimental cages equipped with ovitraps containing either dechlorinated water (control) or BSAW at the calculated LC_50_ for an exposure period of 48 h. Ovitraps were randomly distributed across the cage floor and retrieved every 48 h over a 10-day assay period. The results were quantified using the oviposition deterrence index (ODI), calculated according to the following equation proposed by Quiroz-Martínez et al. [42]:

(1) $$\mathrm{ODI}\;\left(\%\right)=\frac{\left(\mathrm{NT}-\mathrm{NS}\right)}{\left(\mathrm{NT}+\mathrm{NS}\right)}\times 100,$$

where NT represents the total number of eggs found in the BSAW treatment and NS denotes the total number of eggs in the control. The ODI values range from −100 to +100, with negative values indicating deterrence and positive values indicating attractiveness.

### 4.7. Effects of BSAW on the Gut Microbiota Homeostasis

#### 4.7.1. Sample Preparation

To investigate the effects of BSAW on the gut microbiota of the *A. aegypti* larvae (L4), a rigorous sterilization protocol was employed. Initially, the larvae were immersed in 70% ethanol (*v*/*v*), followed by five rinses with Milli-Q water to eliminate surface contaminants (Sigma-Aldrich, St. Louis, MO, USA). The midguts of ten larvae were excised using an 8 mm length, 0.3 mm caliber needle and immediately homogenized in 10 mL of Luria–Bertani broth. This homogenized product was designated as the larval intestinal homogenate (LIH). All procedures were performed under stringent sterile conditions within a biological safety laminar flow cabinet to maintain sample integrity [43].

#### 4.7.2. Impact of BSAW on Microbiota

The effects of BSAW on the bacterial composition within the LIH were assessed using the microdilution method, following the guidelines established by the National Committee for Clinical Laboratory Standards [44]. A volume of 100 µL of LIH was cultured in sterile Mueller–Hinton broth (Kasvi), standardized to an optical density corresponding to a McFarland scale of 0.5. Subsequently, 100 µL of BSAW was added to the wells at a concentration equivalent to LC_50_ for a 48-hour exposure, followed by incubation at 37 °C for 22 h. Following the incubation, 5 µL of a 2,3,5-triphenyl tetrazolium chloride revealing solution (Sigma-Aldrich) was incorporated, and a further incubation was conducted for 2 h. Tetracycline (Prati-Donaduzzi, Toledo, PR, Brazil) served as a control at identical dilution concentrations.

#### 4.7.3. Characterization of the Intestinal Microbiota of *A. aegypti* Larvae

To isolate bacterial colonies from the LIH, the exhaustion-seeding technique was implemented, involving consecutive composite seedings. The isolated colonies were characterized through both macroscopic and microscopic evaluations. Macroscopic characterization was performed using a stereoscope (ECZ-EMB, Ionlab, Bangalore, Karnataka; 10×) to examine essential morphological parameters, including color, shape, elevation, margin, surface, consistency, and size. Colony shapes were visually classified as circular, filamentous, irregular, or punctiform. Elevation was classified as flat, raised, convex, umbonate, crateriform, or acute, while margins were described as entire, undulate, rhizoid, lobate, or filamentous. Surface aspects were classified as smooth, glistening, rough, dull, or rugose, and consistency was evaluated using a bacteriological loop, categorizing colonies as butyrous, viscid, brittle/friable, or mucoid. Colony size was measured using a digital caliper (Series 500, Mitutoyo, Kawasaki, Japan). Cellular morphological characterization was conducted through smear preparation and Gram staining, followed by microscopic analysis using a Nikon Eclipse EI Microscope (Nikon Instruments Inc., Melville, NY, USA) at 100× magnification. Bacterial shape, arrangements, and Gram classification were evaluated according to the methodologies outlined by Bartholomew and Mittwer [45].

## 5. Conclusions

In summary, the innovative approach presented in this study offers a safer and more sustainable alternative within the framework of rhamnolipid production. This method demonstrates significant potential for new industrial applications in the integrated management of epidemiologically significant insect vectors, thereby emphasizing the importance of environmental conservation. It fosters the incorporation of traditional knowledge and practices in the development of bioproducts and bioprocesses, enhancing the cultural relevance and acceptance of biotechnological advancements.

Utilizing agro-industrial waste as a substrate for fermentative processes encourages discussions surrounding sustainable management practices and stimulates innovative solutions that enhance commercial competitiveness in alignment with contemporary sustainability goals. By transforming waste materials into valuable products, the proposed method not only advances the circular economy but also exemplifies the transformative potential of biomass utilization.

Future research endeavors should focus on elucidating the mechanisms of action of BSAW, assessing its effectiveness under field conditions, and exploring its potential for controlling other mosquito species. Investigations into the scalability of BSAW production are warranted, alongside comprehensive assessments of the long-term impacts of its application. Furthermore, exploring strategies for integrating BSAW into existing mosquito control frameworks is crucial, as this could substantially contribute to addressing current public health challenges posed by vector-borne diseases. Ongoing investigations are directed toward evaluating the effects of lower concentrations of BSAW and assessing potential synergistic interactions with additional compounds. These studies hold promise for providing meaningful contributions to the current landscape of vector management.

Ultimately, the findings of this research underscore the critical importance of advanced techniques for biomolecule extraction and utilization, such as the production of rhamnolipids. By aligning research efforts with the realms of biotechnology, sustainability, and public health, this study expands the scientific discourse and lays the groundwork for practical applications that could significantly enhance vector management strategies.

## 6. Patents

The medium formulation for BSAW production is safeguarded under industrial confidentiality, with an Invention Patent filed (BR1020230146740) at the Brazilian Institute of Industrial Property.

## Figures and Tables

**Table 1:** Physicochemical properties of biosurfactant (BSAW) produced by *Pseudomonas aeruginosa* BM02.

Compounds	Emulsification Index (%)	Surface Tension (mN/m)	Interfacial Tension (mN/m)
BSAW	59.30 ± 1.00	28.52 ± 0.22	3.27 ± 0.10
VSM	0.00 ± 0.00	69.90 ± 0.74	17.00 ± 0.30
SDS	69.90 ± 1.10	Nt	Nt

VSM: Vegetable saline medium (negative control for surface and interfacial tension assays); SDS: 1% Sodium dodecyl sulfate (positive control for emulsification assays); Nt: Not tested; Results are expressed as mean ± standard deviation (n = 3).

**Table 2:** Proximate composition of andiroba biomass wastes.

Parameter	Values (%)
Total lipids	57.02 ± 0.58
Carbohydrates	25.06 ± 0.46
Crude protein	10.76 ± 0.23
Ash	4.42 ± 0.08
Moisture	2.75 ± 0.03

Results are expressed as mean ± standard deviation (n = 3).

**Table 3:** Lethal concentration values for *Aedes aegypti* larvae exposed to biosurfactant (BSAW) produced by *Pseudomonas aeruginosa* BM02.

Lethal Concentration (LC)		Slope (SE)	χ^2^	R^2^
24 h post-exposure				
LC_50_	4.656 (1.448–14.967)	1.256 (0.259)	0.143	0.908
LC_90_	47.792 (14.865–153.648)			
LC_99_	319.075 (99.247–1025.812)			
48 h post-exposure				
LC_50_	1.189 (0.480–2.944)	1.253 (0.201)	0.035	0.883
LC_90_	14.534 (5.868–35.998)			
LC_99_	119.893 (45.175–277.145)			

LC_50_, LC_90,_ and LC_99_: Lethal concentration, LC, for 50, 90, and 99% of larvae, expressed in mg mL^−1^ of BSAW (lower–upper).

**Table 4:** Oviposition profile of *Aedes aegypti* and oviposition deterrence index for BSAW (biosurfactant from andiroba wastes).

Oviposition Profile (Days Post-Exposure)	BSAW	Control
2	0 (0–0) ± 0.00	0 (0–1) ± 0.58
4	2 (0–7) ± 4.04	90 (0–169) ± 85.10
6	7 (3–16) ± 7.51	87 (62–117) ± 27.75
8	4 (0–10) ± 5.51	30 (3–48) ± 23.63
Total eggs	41	624
**ODI = −79%**		

The oviposition profile is expressed as the mean (lower–upper) ± standard deviation (n = 3).

## Data Availability

All data needed to evaluate the conclusions in the paper are included in the paper and Supplementary Materials. Additional data related to this paper are available from the corresponding authors upon request.

## Supplementary Materials

The following supporting information can be downloaded at: https://www.mdpi.com/article/10.3390/molecules30030618/s1, Table S1: Macro and microscopic characterization of bacterial composition within the intestinal homogenate of *Aedes aegypti* larvae (L4); Figure S1: ESI-MS (negative mode, 0 to 1000 *m/z*, HPLC-grade methanol) spectrum for rhamnolipid congeners from BSAW.
