# Supplementary material for: Pseudomonas aeruginosa Rhamnolipids Produced by Andiroba (Carapa guianensis Aubl.) (Sapindales: Meliaceae) Biomass Waste from Amazon: A Potential Weapon Against Aedes aegypti L. (Diptera: Culicidae)
Source: Molecules. 2025 Jan 31;30(3):618. doi: 10.3390/molecules30030618 (PMC11821126; doi:10.3390/molecules30030618)

Supplementary Materials

Pseudomonas aeruginosa Rhamnolipids Produced by Andiroba (*Carapa guianensis* Aubl.) (Sapindales: Meliaceae) Biomass Waste from Amazon: A Potential Weapon Against *Aedes aegypti* L. (Diptera: Culicidae)

Giulian César da Silva Sá, Pedro Vitor Vale Bezerra, Evelly Oliveira Ramos, Alexandre Orsato, Karoline Leite, Alan Moura Feio, Lucas Mariano Siqueira Pimentel, Joane de Almeida Alves, Glenda Soares Gomes, Pamela Dias Rodrigues, Cristina M. Quintella, Sinara Pereira Fragoso, Emilly Cruz da Silva, Adriana Ferreira Uchôa, Sidnei Cerqueira dos Santos

Table S1. Macro and microscopic characterization of bacterial composition within the intestinal homogenate of *Aedes aegypti* larvae (L4).

Macroscopic characterization of the colonies

Strain	Chromogenesis	Shape	Elevation	Margin/Edges	Surface	Consistency	Size
1 (Fig. 3A)	White	Irregular	Umbonate	Undulate (Wavy)	Rough	Butyrous	3mm
2 (Fig. 3B)	White	Irregular	Umbonate	Undulate (Wavy)	Pleated	Mucoid	3mm
3 (Fig. 3C)	Light Green	Circular	Flat	Entire (Smooth)	Smooth/Shiny	Viscid	<2mm
4 (Fig. 3D)	Beige	Circular	Flat	Entire (Smooth)	Smooth/Shiny	Viscid	2mm
5 (Fig. 3E)	Beige	Circular	Flat	Entire (Smooth)	Smooth/Shiny	Viscid	2mm
6 (Fig. 3F)	Light Green	Circular	Flat	Entire (Smooth)	Smooth/Shiny	Viscid	<2mm

Microscopic characterization of the cells

Strain	morphology	Arrangement	Gram
1 (Fig. 3G)	Rod-shaped - Bacilli (long)	Isolated, diplobacillus, streptobacillus	Positive
2 (Fig. 3H)	Bacilli (long)	Isolated, diplobacillus, streptobacillus	Positive
3 (Fig. 3I)	Spherical - Cocci	Isolated, diplococcus, staphylococcus	Positive
4 (Fig. 3J)	Rod-shaped - Bacilli (short)	Isolated, diplobacillus	Negative
5 (Fig. 3K)	Rod-shaped - Bacilli (short)	Isolated, diplobacillus	Negative
6 (Fig. 3L)	Spherical - Cocci	Isolated, diplococcus, staphylococcus	Positive

Strains 1-4: Intestinal homogenate (control); Strains 5-6: Intestinal homogenate (treatment); BSAW concentration: 1.0 mg/mL

Figure S1. ESI-MS (negative mode, 0 to 1000 *m/z*, HPLC-grade methanol) spectrum for rhamnolipid congeners from BSAW.

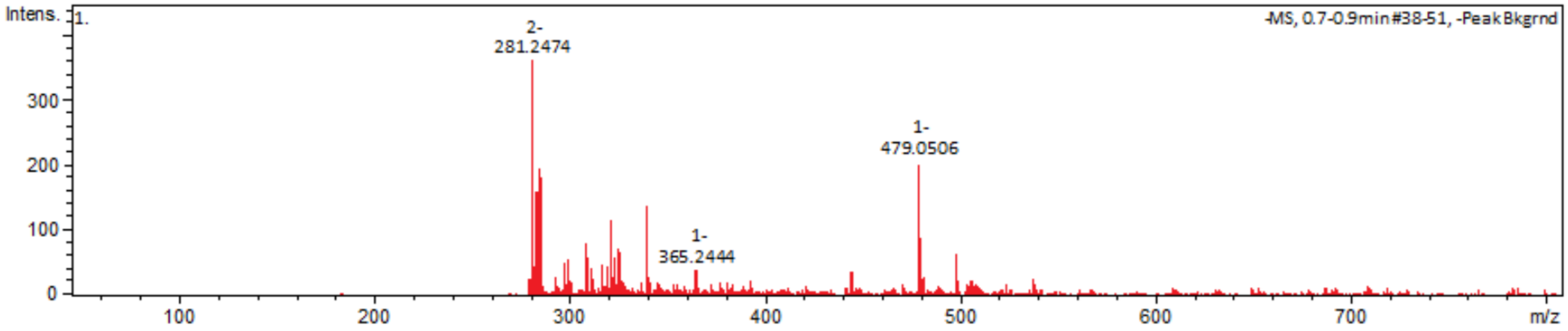

Supplement: Supplementary file 1 [file molecules-30-00618-s001.zip › molecules-3396246-supplementary.pdf]
